# Assessing handwriting skills in a web browser: Development and validation of an automated online test in Japanese Kanji

**DOI:** 10.3758/s13428-024-02562-6

**Published:** 2024-12-30

**Authors:** Tomohiro Inoue, Yucan Chen, Toshio Ohyanagi

**Affiliations:** 1 Department of Psychology, The Chinese University of Hong Kong, Hong Kong SAR, China; 2 Department of Liberal Arts and Sciences, Sapporo Medical University, Sapporo, Japan

**Keywords:** Handwriting, Automated online assessment, Convolutional neural network, Japanese Kanji

## Abstract

Online language and literacy assessments have become prevalent in research and practice across settings. However, a notable exception is the assessment of handwriting and spelling, which has traditionally been conducted in person with paper and pencil. In light of this, we developed an automated, browser-based handwriting test application (*Online Assessment of Handwriting and Spelling:* *OAHaS*) for Japanese Kanji (Study 1) and examined its psychometric properties (Study 2). The automated scoring function using convolutional neural network (CNN) models achieved high recall (98.7%) and specificity (84.4%), as well as high agreement with manual scoring (95.4%). Additionally, behavioral validation with data from primary school children (*N* = 261, 49.0% female, age range = 6–12 years) indicated the high reliability and validity of our online test application, with a strong correlation between children’s scores on the online and paper-based tests (*r* = .86). Moreover, our analysis indicated the potential utility of writing fluency measures (latency and duration) that are automatically recorded by OAHaS. Taken together, our browser-based application demonstrated the feasibility and viability of remote and automated assessment of handwriting skills, providing a streamlined approach to research and practice on handwriting. The source code of the application and supporting materials are available on Open Science Framework (https://osf.io/gver2/).

**Supplementary information:**

The online version contains supplementary material available at 10.3758/s13428-024-02562-6.

## Introduction

In recent years, online assessments of children’s language and literacy skills, such as vocabulary knowledge and word reading, have become increasingly prevalent in research and practice in psychology, education, and speech-language pathology (e.g., Hautala et al., 2020; Hoskins et al., 2021; Manning et al., 2020; Varga et al., 2022; Yeatman et al., 2021). Several online screening tools for language and literacy difficulties (e.g., dyslexia, developmental language disorder) have also appeared (Asselborn et al., 2018; Guinet & Kander, 2010; Hulme et al., 2024; Hurford & Wines, 2021; Zugarramurdi et al., 2022), and this trend seems to be accelerating in response to the COVID pandemic worldwide (Antoniou et al., 2022; Castilla-Earls et al., 2022; Ho et al., 2023). To date, however, there is a paucity of evidence regarding the psychometric properties (i.e., reliability and validity) of online language and literacy assessments. This raises questions about their applicability to scientific research and clinical practice (for relevant discussions, see Antoniou et al., 2022; Magimairaj et al., 2022; Peña & Sutherland, 2022). This problem is particularly salient for handwriting and spelling assessments, which are typically administered in person with paper and pencil (e.g., Ho et al., 2007; Wechsler, 2001; Wilkinson & Robertson, 2006, for standardized tests of handwriting).

To address these issues, we developed a self-administered, browser-based handwriting assessment for Japanese Kanji characters (Study 1), which we call the *Online Assessment of Handwriting and Spelling* (*OAHaS*). We implemented an automated scoring function in OAHaS by using convolutional neural network (CNN) models for image classification. This allowed us to overcome the methodological problems that are inherent in the manual scoring of handwritten responses (see below for a review). Importantly, we used a language-independent image classification technique in the automated scoring function to make the test development framework applicable to any language and script. In addition, we evaluated the psychometric properties of OAHaS through a behavioral validation study with data obtained from primary school children (Study 2). In doing so, we sought to demonstrate the feasibility and validity of online handwriting assessment and to provide an effective tool for researchers and practitioners focused on understanding and improving handwriting and spelling skills, especially in children.

In the following, we will first provide a brief overview of the characteristics of traditional handwriting assessment tools, both paper-based and digitized, and highlight their methodological limitations. We will then describe how an automated handwriting assessment can overcome these limitations and achieve greater applicability in both scientific research and clinical practice in various fields, including psychology, education, and speech-language pathology.

### Traditional handwriting assessment

Traditional assessments of handwriting and spelling skills have typically been administered in a paper-and-pencil format, requiring test-takers (e.g., children) to write answers (e.g., letters, words, sentences) on a response sheet (for a review, see Kohnen et al., 2009). For example, in the Spelling subtest of the Wide Range Achievement Test (WRAT; Wilkinson & Robertson, 2006), a standardized test battery of academic achievement in English, the examiner first reads a target word aloud and then asks the test-taker to write a dictated letter or word on a response sheet. Similarly, the Spelling subtest of the Wechsler Individual Achievement Test (WIAT; Wechsler, 2001) measures children’s handwriting of single sounds and words from dictation in a paper-and-pencil format. This testing format has been widely used in standardized assessments of handwriting and spelling across various languages and cultures (e.g., Dutch: Geelhoed & Reitsma, 1999; French: Wechsler, 2005; German: Moll & Landerl, 2010), including morphographic scripts of Chinese and Japanese Kanji (Chinese: Ho et al., 2007; Japanese: Uno et al., 2017). For example, in the Chinese Word Dictation subtest of the Hong Kong Test of Specific Learning Disabilities in Reading and Writing (HKT-SpLD; Ho et al., 2007), the examiner reads aloud two-character Chinese words of different difficulty levels (e.g., 朋友 ‘friend’, 公園 ‘park’) and asks the test-taker to write the answer on a response sheet.

Paper-based standardized tests have also been used in studies on children’s handwriting development and disabilities (e.g., Desrochers et al., 2018; Georgiou et al., 2020; Graham et al., 2000; Ho et al., 2004; Kim et al., 2014). Some researchers have also developed their own measures of handwriting and spelling and used them in their studies (e.g., Inoue et al., 2017, 2022; Mouzaki et al., 2007; Yang et al., 2022; Ye et al., 2022). It should be noted, however, that these handwriting tests have several important methodological limitations. First, paper-based tests, whether standardized or researcher-developed, capture only the final written responses and do not provide information about the handwriting processes, such as latency, movement duration, and stroke order (see Asselborn et al., 2018; Rosenblum et al., 2004, for relevant discussions). Several studies have shown that children’s handwriting fluency, often operationalized with writing latency and duration, plays an important role in their higher-level writing processes, such as transcription (Limpo et al., 2017; Skar et al., 2022). Furthermore, the information on stroke order is of particular importance for morphographic writing systems such as Chinese and Japanese Kanji, where correct stroke order has been demonstrated to influence actual handwriting performance levels (e.g., Hsiung et al., 2017; Xu et al., 2020). In light of these findings and the evidence supporting the role of motor learning in handwriting development (e.g., Kandel & Perret, 2015; Rosenblum et al., 2004; Tseng & Hsueh, 1997), it is evident that these performance indicators are crucial for a more precise and informative assessment of handwriting processes.

A second limitation of paper-based handwriting assessment is the lower reliability of manual scoring. In fact, it is often subject to scorer bias, where there is often a discrepancy in judgment between scorers. Even when prespecified scoring criteria are employed, as is the case in the majority of standardized assessments (see Ho et al., 2007; Wechsler, 2001), scorers require considerable training to evaluate handwriting responses with substantial interindividual variability. Although empirical studies rarely address this issue, some researchers have attempted to address it by calculating interrater agreement rates (i.e., interrater reliability). However, in most cases, interrater reliability is far from perfect (typically around .70–.80; e.g., Hamstra-Bletz & Blöte, 1990), which raises questions about the reliability of the scoring. In other words, when a traditional paper-based handwriting assessment with manual scoring is employed, a considerable amount of random error is likely to be introduced into the measured data. This error can subsequently obscure the true relationship between the variables of interest, resulting in biased estimated associations (Bollen, 1989; Carroll et al., 2006).

Finally, a third limitation is that the scoring of handwriting responses in paper-based tests must be conducted by a human scorer, either immediately after each response or collectively after all responses have been made. This is often a highly labor-intensive and time-consuming process, and it is likely to be one factor contributing to the lower reliability of the scoring. To illustrate, the Spelling subtest of the Woodcock-Johnson III Test of Achievement (Woodcock et al., 2001) consists of 59 items; although test-takers are not always required to answer all items due to the discontinuation rule (i.e., the test is terminated if a child answers six consecutive questions incorrectly), if researchers administer an average of 40 items to 500 children, for example, a total of approximately 20,000 manual judgments are required. Not surprisingly, such a large number of repeated scorings is highly prone to human error and fluctuating scoring criteria, introducing additional measurement errors into the data. This problem is likely to be more serious in a large-scale research project or national survey with many thousands of test-takers.

### Digitized handwriting assessment

Some researchers have used electronic devices (e.g., digitizers, tablet computers) to examine the characteristics of handwriting processes by evaluating various performance indicators, such as latency and duration (e.g., Alamargot et al., 2006; Gosse et al., 2021; Huang et al., 2021; Kandel & Perret, 2015; Rosenblum et al., 2004; Wang et al., 2020). For example, Kandel and Perret (2015) examined different processing levels involved in handwriting among French-speaking children in Grades 3 and 4. They used a digitizer to measure response latency, movement duration, and fluency and found that word frequency and regularity affected response latency. Similarly, in a study with Chinese university students, Wang et al. (2020) used a digitizer to evaluate several aspects of handwriting performance, including accuracy, latency (an indicator of orthographic access), and duration (an indicator of motor execution). Their results showed that word frequency, age of acquisition, and word context (in which a character appears) all predicted the accuracy, latency, and duration of Chinese handwriting.

Although these studies have enhanced our understanding of the processes underlying handwriting and spelling, none of them incorporated automated scoring of handwriting responses in the measurement. Consequently, the aforementioned methodological issues of manual scoring (i.e., lower reliability) remain unresolved. In fact, the measures used in these studies were designed for psychological experiments, and their application outside the laboratory setting was beyond their scope. As we demonstrate in the two studies below, an automated scoring function using convolutional neural network (CNN) models for image classification offers a solution for evaluating handwriting skills among a group of test-takers in more ecologically valid contexts (e.g., clinics, schools, homes).

### Convolutional neural network (CNN) models for image classification

CNN models have become the mainstream image classification algorithm due to their promising performance and have been widely used for automated image classification, including that of handwriting (e.g., Altwaijry & Al-Turaiki, 2021; Corbillé et al., 2020; Kaur & Gandhi, 2020). Unlike traditional machine learning models that require manual feature extraction, CNNs can automatically learn hierarchical features from input, eliminating the need for explicit and complex feature extraction (Ponti et al., 2017). Previous studies have demonstrated the efficacy of CNNs in extracting relevant features from handwritten samples, outperforming traditional feature extraction methods (e.g., Jasira et al., 2023; Rahmanian & Shayegan, 2021; Zamani et al., 2015). For example, in a study using handwriting samples to screen children for dyslexia, Jasira et al. (2023) compared CNN models with machine learning models (support vector machine [SVM] and random forest) and showed that their trained CNN model achieved higher classification performance (with accuracy of .95, precision of .97, recall of .93, and F1-measure of .95) than the SVM (with accuracy of .88, precision of .91, recall of .82, and F1-measure of .86) and random forest (with accuracy of .92, precision of .94, recall of .89, and F1-measure of .95).

This advanced feature extraction capability of CNNs would help classify Japanese Kanji characters, which have high configurational complexity (Chang et al., 2018). In fact, for handwritten Kanji, even small variations in stroke length can significantly affect the accuracy of the written character, which can lead to misclassification of written characters by the models (as we will illustrate in Study 1 below). With CNNs, we can better address the challenges posed by different handwriting styles, such as “loose” or “tight” writing by young children (i.e., two components of the character are far apart or too close together) and subtle variations in stroke length. This helps ensure reliable scoring in our automated handwriting assessment.

In the present study, three advanced CNN models were selected for the classification of correct and incorrect handwritten Kanji characters: Xception (Chollet, 2017), Inception V3 (Szegedy et al., 2016), and ResNet50 (He et al., 2016). Notably, all three models have demonstrated excellent handwriting recognition performance for relatively complex scripts such as Indic languages (e.g., Jiang, 2020; Mhapsekar et al., 2020). For example, Xception and Inception V3 achieved 98.2% and 98.5% classification accuracy, respectively, for Urdu handwriting recognition (Jiang, 2020). Similarly, ResNet50 achieved 99.35% accuracy in Devanagari handwriting recognition, outperforming the state-of-the-art recognition models in that language (Mhapsekar et al., 2020). The outstanding performance of the three models in these languages underscores their suitability for our study and reinforces their potential for achieving accurate classification results for Japanese Kanji characters.

### The present studies

Here, we report on two studies in which we developed and validated a self-administered, automated, browser-based handwriting test (*Online Assessment of Handwriting and Spelling:* *OAHaS*) for Japanese Kanji. Kanji is a morphographic script originated from Chinese in which each character can represent multiple sounds and morphemes depending on the word context (e.g., 空 can mean ‘sky’ and ‘empty’, and it can be read as /sora/, /kuu/, /a/, and /kara/). A total of 2136 Kanji characters are generally used in modern Japanese text (Taylor & Taylor, 2014), and children learn a total of 1026 characters as part of the national curriculum in primary education (for a more detailed description of the characteristics of Japanese Kanji, see Taylor & Taylor, 2014).

In Study 1, we first developed a browser-based application for collecting handwriting samples of Kanji characters, and then, by using the handwriting samples from Japanese primary school children, adolescents, and adults, we developed convolutional neural network (CNN) models for image classification. We then evaluated the classification performance of the models. To the best of our knowledge, this is among the first studies to apply CNN models for image classification in the automatic scoring of handwriting assessments.

In Study 2, we investigated the psychometric properties (i.e., reliability and validity) of OAHaS as a handwriting assessment for children through behavioral validation. Specifically, we assessed a sample of Japanese primary school children in Grades 1 to 6 on their Kanji handwriting skills using both OAHaS and a traditional paper-based test. In addition, we examined the potential utility of two response time measures (writing latency and duration) automatically recorded by OAHaS as indicators of children’s handwriting fluency. Through these two studies, we discuss the feasibility and validity of the web-based handwriting assessment application developed here (OAHaS) for research and clinical practice in various fields, as well as its potential for extension to other languages and writing systems.

## Study 1: Development of online handwriting test

We developed the automated online Kanji handwriting test (OAHaS) in the following three steps. First, to collect handwriting samples to develop CNN models, we developed a separate web-based application, *Handwriting Sample Collector Application* (*HaSCAp*). Second, we constructed three CNN models (Xception, Inception V3, and ResNet50) for image classification of handwritten Kanji characters, which were then incorporated in OAHaS for automated scoring. Finally, we evaluated the classification performance of the CNN models. We compared the scoring performance of our models with that of a free online handwriting recognition service provided by Google.

### Materials and system

#### System overview

**Fig. 1:**
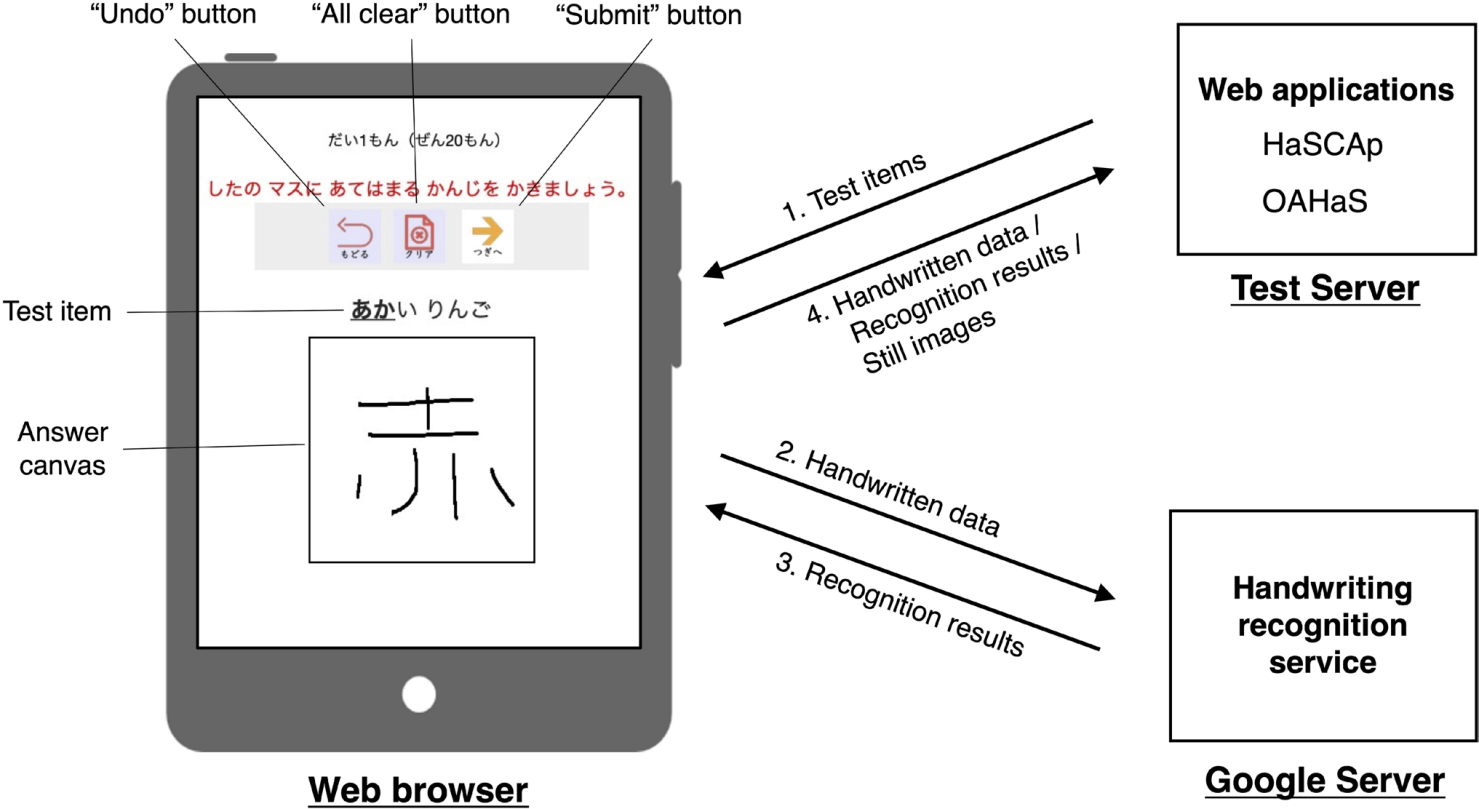
System overview of the online handwriting applications developed in the study. *Note.* For the details of the system overview, see System Overview in Study 1. HaSCAp = Handwriting Sample Collector Application; OAHaS = Online Assessment of Handwriting and Spelling

Figure 1 shows the system overview of our web-based handwriting applications, HaSCAp and OAHaS. Both applications were developed using CakePHP, an open-source web development framework for PHP. In addition to PHP, HTML, CSS, and JavaScript were used in the development. All source codes have been made available on OSF at https://osf.io/gver2/.

As shown in Fig. 1, the participants accessed the test server located in the lab at Sapporo Medical University to start the applications using a web browser. Brief instructions written in syllabic Hiragana characters in Japanese were presented in the browser to indicate test items; the participants were required to read the sentence and write the Kanji character specified by an underline in each sentence (e.g., “あかい りんご”)^1^ in the canvas area using an electronic pen or their finger. Then, by clicking the “Submit” button, the handwritten data (including XY coordinate information of each stroke, stroke order, and temporal information for strokes such as latency and duration) was sent to Google’s Japanese Kanji recognition server to obtain a recognition result of candidate Kanji characters.^2^ We developed a JavaScript program to use their service by sending the handwritten data of Kanji characters in the answer canvas to the following URL in our web applications (https://inputtools.google.com/request?itc=ja-t-i0-handwrit&app=demopage). The applications then received ten candidates of characters or texts for the sent handwritten data. After receiving recognition results from Google’s server, the handwritten data, recognition results, and still images of the handwritten Kanji characters were submitted to the test server to be stored in the database.

#### Step 1: Collecting handwriting samples for model development

We collected handwriting samples for 120 Kanji characters (20 characters each for Grades 1 to 6 selected from the national curriculum; Ministry of Education, Culture, Sports, Science and Technology, 2017; see Appendix A) using HaSCAp. The characters were adapted from a paper-based Kanji writing test developed in a previous project on literacy development in Japanese children (e.g., Inoue et al., 2017, 2022). The participants consisted of 177 Japanese speakers of different ages, primarily school-age children, as shown in Table 1. The participants in Grades 1 to 6 (*n* = 125) were asked to write a subset of characters corresponding to their grade level (20 characters each); the participants in Grade 7 and above (*n* = 52) were asked to write all 120 characters. We included participants beyond primary school age to ensure sufficient handwriting samples with greater variability for the model development. A trained human evaluator with expertise in literacy assessment scored all collected samples to determine whether they were correct/incorrect; the total number of correct answers was 7133 (the numbers of correct answers for each item are presented in Appendix A). The dataset of all correct answers, termed *Dataset 1*, was then used to develop CNN models for the automatic scoring of handwritten Kanji characters.

**Table 1:** Sample sizes for each web-based handwriting application

	Study 1 (HaSCAp)		Study 2 (OAHaS)	
Grade	*N*	%	*N*	%
1	31	(17.5%)	35	(13.4%)
2	14	(7.9%)	48	(18.4%)
3	28	(15.8%)	37	(14.2%)
4	18	(10.2%)	44	(16.9%)
5	20	(11.3%)	54	(20.7%)
6	14	(7.9%)	43	(16.5%)
7–9	6	(3.4%)	–	–
10–12	6	(3.4%)	–	–
13 or above	40	(22.6%)	–	–
Total	177		261	

*Note.* HaSCAp = Handwriting Sample Collector Application; OAHaS = Online Assessment of Handwriting and Spelling

Next, we conducted a closer inspection of the incorrect answers to identify how participants were prone to make mistakes in writing Kanji characters. This allowed us to identify two common types of mistakes: (1) orthographic errors (e.g., incorrect characters with extra/missing strokes or stroke intersections; see Appendix B); (2) semantic/homophone errors (e.g., existent Kanji characters that do not match the word context; see Appendix C). In identifying incorrect characters with orthographic errors, we followed the criteria used in a national survey (Synthetic Research Institute of Elementary Education, 2005, pp. 59–62). Specifically, we checked whether (a) there were extra or missing strokes, (b) the strokes were not too long or too short, (c) the strokes were connected or separated, and (d) the strokes intersected or did not intersect. Prior work has suggested that incorporating these variations in the sample sets for CNN model development could prevent over-fitting and further enhance classification performance (e.g., Dutta et al., 2018). Accordingly, we prepared two additional sets of handwriting samples including these common types of errors, termed *Dataset 2* and *Dataset 3*, respectively, for the development of our CNN models capable of recognizing such variations. Image files of incorrect characters with orthographic errors in Dataset 2 (*N* = 1023) were manually created from the corresponding correct characters in Dataset 1 by modifying strokes (e.g., adding, deleting, changing the length; see Appendix B). In turn, image files of existent Kanji characters in Dataset 3 (*N* = 2422) were taken from the Electrotechnical Laboratory (ETL) Character Database (http://etlcdb.db.aist.go.jp/). In total, 10,578 handwriting samples in the three datasets (Datasets 1 to 3) were used for the CNN model development. Each handwriting image file in the datasets was a monochrome JPEG file. All of the datasets are freely available at https://osf.io/gver2/.

#### Step 2: development of convolutional neural network (CNN) models

**Fig. 2:**
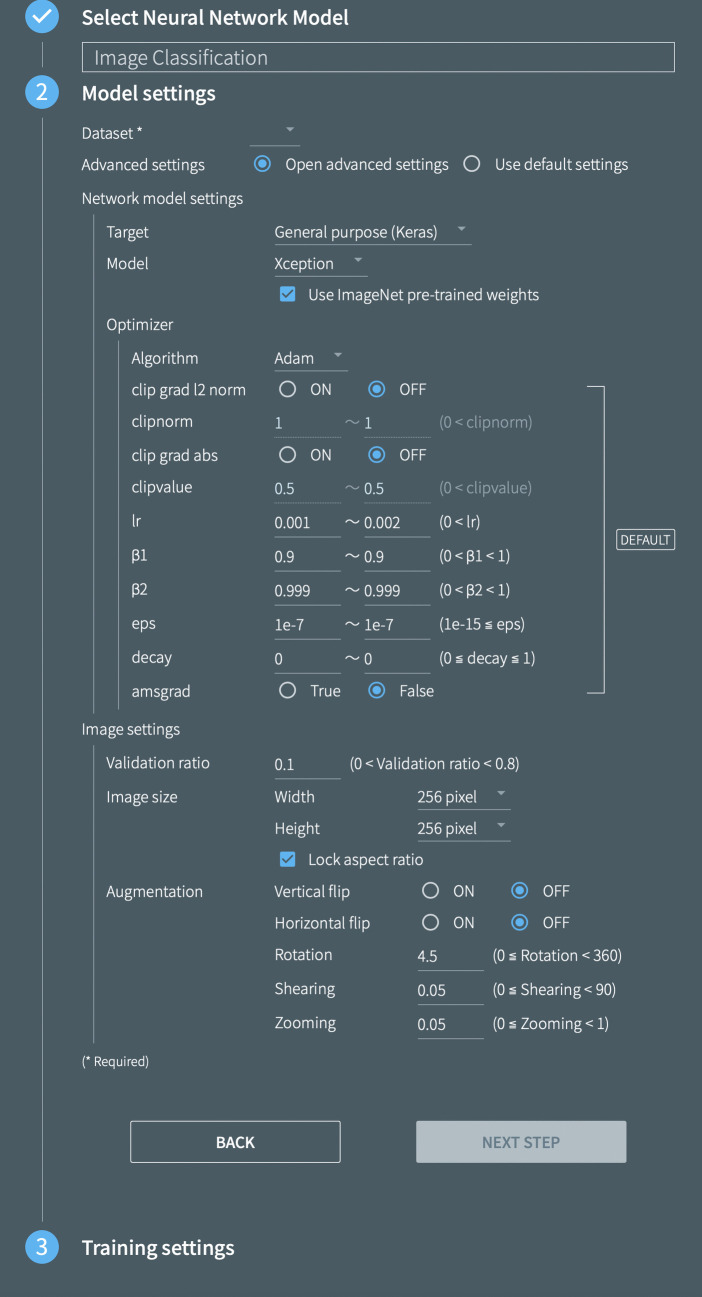
Model settings panel in Deep Analyzer (GHELIA Inc., Japan). *Note*. The model parameter settings in Deep Analyzer (GHELIA Inc., Japan) are shown. The base models (Xception, Inception V3, and ResNet50) were selected in the “Model” pulldown menu

We used a package software called *Deep Analyzer* (GHELIA Inc., Japan) to build our CNN models. Figure 2 shows the parameter settings in Deep Analyzer for the development of the models. In this study, we used Xception (Chollet, 2017), Inception V3 (Szegedy et al., 2016), and ResNet50 (He et al., 2016) as base models. The three models have been widely used in previous studies, showing their promising performance in image classification (e.g., Altwaijry & Al-Turaiki, 2021; Corbillé et al., 2020; Kaur & Gandhi, 2020). The epoch number was set to 100. In order to evaluate the impact of incorporating common handwriting errors in model training, we developed four separate CNN models for each base model, with the following combinations of training datasets: (1) Dataset 1 (correct characters) alone, (2) Dataset 1 and Dataset 2 (orthographic errors), (3) Dataset 1 and Dataset 3 (semantic/homophone errors), and (4) all three datasets.

#### Step 3. Evaluation of the classification performance

To assess the classification performance of our CNN models and the Google handwriting recognition service, we employed five commonly used metrics: accuracy, precision, recall, F-measure, and specificity. Table 2 shows the confusion matrix, and the five metrics were calculated in the following equations:

**Table 2:** Confusion matrix for the evaluation of model performance

Label	Inference (Prediction)	
	Negative (incorrect answer)	Positive (correct answer)
Negative (incorrect answer)	True negative (TN)	False positive (FP)
Positive (correct answer)	False negative (FN)	True positive (TP)

$$\begin{array}{l}Accuracy= \frac{TP+TN}{TP+FP+TN+FN}\\ Precision=\frac{TP}{TP+FP}\\ \begin{array}{l}Recall=\frac{TP}{TP+FN}\\ F-measure=2\times \frac{Precision\times Recall}{Precision+Recall}\\ Specificity=\frac{TN}{TN+FP}\end{array}\end{array}$$

Furthermore, to assess the generalizability of the models’ performance beyond the training data, we evaluated the classification accuracy of the models on independent sets of handwriting samples. These datasets were originally collected in a previous study with Japanese children in Grades 1 and 2 using a paper-based writing test (*N* = 153 and 135, respectively; see Inoue et al., 2017, 2022, for details about their samples). For this purpose, we created separate sets of image files of handwriting samples for each grade, termed *g1f* (Grade 1 Fall) and *g2s* (Grade 2 Spring), respectively, by scanning the answer sheets of the paper-based Kanji writing test (see Appendix D). We then tested the classification performance of the models on these additional datasets.

### Results

Table 3 presents the calculated scores (in percentages) for the five performance metrics (accuracy, precision, recall, F1-measure, and specificity) for the three base models (Xception, Inception V3, and ResNet50). When only Dataset 1 (correct characters) was used for model development, the classification performance yielded relatively lower values for accuracy (90.64% to 92.09%), F-measure (94.17% to 95.17%), and specificity (67.19% to 71.99%) compared to the other conditions in all three base models.

**Table 3:** Discriminant accuracy, precision, recall, f-measure, and specificity values for the convolutional neural network models

	Match			Mismatch							
Model and Dataset	TP	TN	Total	FN	FP	Total	Accuracy	Precision	Recall	F-measure	Specificity
Xception											
Dataset 1	2633	475	3108	35	232	267	92.09	91.90	98.69	95.17	67.19
Dataset 1+2	2639	574	3213	29	133	162	95.20	95.20	98.91	97.02	81.19
Dataset 1+3	2659	482	3141	9	225	234	93.07	92.20	99.66	95.79	68.18
Dataset 1+2+3	2633	587	3220	35	120	155	95.41	95.64	98.69	97.14	83.03
Inception V3											
Dataset 1	2550	509	3059	118	198	316	90.64	92.79	95.58	94.17	71.99
Dataset 1+2	2592	585	3177	76	122	198	94.13	95.50	97.15	96.32	82.74
Dataset 1+3	2637	485	3122	31	222	253	92.50	92.24	98.84	95.42	68.60
Dataset 1+2+3	2614	597	3211	54	110	164	95.14	95.96	97.98	96.96	84.44
ResNet50											
Dataset 1	2635	494	3129	33	213	246	92.71	92.52	98.76	95.54	69.87
Dataset 1+2	2632	560	3192	36	147	183	94.58	94.71	98.65	96.64	79.21
Dataset 1+3	2654	482	3136	14	225	239	92.92	92.18	99.48	95.69	68.18
Dataset 1+2+3	2628	574	3202	40	133	173	94.87	95.18	98.50	96.81	81.19

*Note.* The first column indicates the base model and the datasets used to develop the model. TP = true positive; TN = true negative; FN = false negative; FP = false positive

The addition of Dataset 2 (orthographic errors) to Dataset 1 resulted in substantial improvements in accuracy (94.13% to 95.20%), F1-measure (96.32% to 97.02%), and specificity (79.21% to 82.74%) compared to using Dataset 1 alone. In comparison, combining Dataset 3 (semantic/homophone errors) with Dataset 1 increased accuracy (92.50% to 93.07%) and F1-measure (95.42% to 95.79%) to a lesser extent than combining Dataset 2, although still higher than the models with Dataset 1 alone.

Most importantly, the models developed using all three datasets together yielded the highest scores on all five metrics compared to the other dataset combinations or Dataset 1 alone, indicating peak classification performance: accuracy (94.87% to 95.41%), precision (95.18% to 95.96%), F-measure (96.81% to 97.14%), and specificity (81.19% to 84.44%). These results were consistently higher than those of Google’s handwriting recognition service, with accuracy (92.92% to 93.99%), precision (92.04% to 94.16%), F-measure (95.70% to 96.28%), and specificity (67.47% to 76.94%) (see Appendix E). Among these metrics, the difference in specificity was particularly pronounced (up to 16.97% higher than Google’s recognition service), indicating that our CNN models were better at filtering out incorrectly written or non-Kanji characters than Google’s handwriting recognition service.

**Fig. 3:**
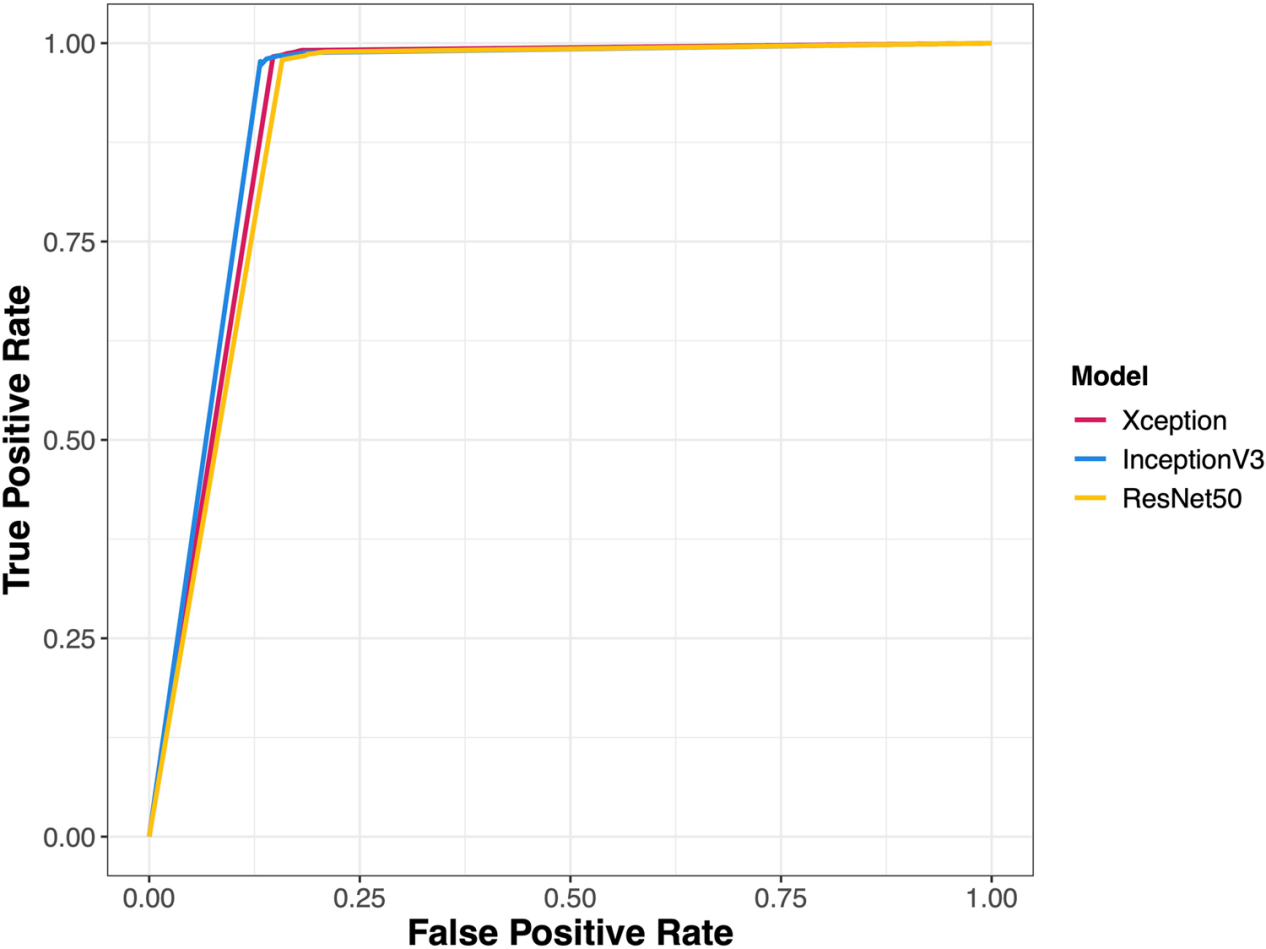
Receiver operating characteristic (ROC) curves for the convolutional neural network models developed in the study. *Note.* The ROC curves for the models trained with all three datasets are shown. The area under the curve (AUC) values are .921 for Xception, .927 for Inception V3, and .914 for ResNet50

Figure 3 presents the receiver operating characteristic (ROC) curves of the three models developed using all datasets. All three models showed excellent classification performance, with the area under the curve (AUC) values of .921 for Xception, .927 for Inception V3, and .914 for ResNet50. Although our study did not aim to compare these three models, Inception V3 seemed to demonstrate relatively superior classification performance with a higher detection rate and a lower false alarm rate compared to Xception and ResNet50.

Finally, we assessed the generalizability of the models’ performance beyond the training data using independent handwriting samples (g1f and g2s). The results are presented in Appendices F and G, respectively. The models trained with all three datasets showed the highest accuracy (92.78% to 96.46%), precision (94.62% to 97.75%), F-measure (95.90% to 98.04%), and specificity scores (62.27% to 80.93%) across datasets, providing evidence for the generalizability of the models. Taken together, these results consistently demonstrate that the CNN models developed here, especially Inception V3, show excellent performance in image classification accuracy for handwritten Kanji characters and can be applied to the automated scoring function in the web-based handwriting test.

## Study 2: Behavioral validation

In Study 2, we investigated the psychometric properties (i.e., reliability and validity) of OAHaS through behavioral validation with a sample of Japanese primary school children. The purpose of the study was to examine (a) the internal consistency of the scores obtained with OAHaS using the automated scoring function, (b) the convergent validity of OAHaS with a traditional paper-based test, and (c) the potential utility of handwriting fluency measures (writing latency and duration) recorded with OAHaS as information about handwriting processes.

### Method

#### Participants

Participants were 261 Japanese children in Grades 1 to 6 (see Table 1; age range = 6–12 years, 49.0% female). They were recruited on a voluntary basis by sending letters of information to the parents of all children in the participating school. All children were native speakers of Japanese, and none had any intellectual, sensory, or behavioral difficulties (based on parents’ reports). The data collection was conducted in accordance with the ethical standards of the American Psychological Association (American Psychological Association, 2017). Ethics approval was also obtained from the Survey and Behavioural Research Ethics Committee of The Chinese University of Hong Kong (Reference No. SBRE-20–633). Parents’ written consent and children’s assent were obtained prior to testing.

#### Materials and procedure

The online handwriting test developed in Study 1 (OAHaS) and a traditional paper-based test of Kanji handwriting were administered. The items for both tests were adapted from the 120 characters (20 characters from each grade 1 to 6) in a researcher-developed handwriting test (e.g., Inoue et al., 2017, 2022; Appendix A). The items were arranged in increasing difficulty based on the percentage of correct answers for each character in a national survey (Japan Foundation for Educational and Cultural Research, 1998). Of the 120 items, the odd-numbered items were used in OAHaS (see Appendix H), while the even-numbered items were used in a paper-based test. This selection procedure ensured an equal distribution of item difficulty across both test formats. The items for each grade were selected based on the national curriculum (Ministry of Education, Culture, Sports, Science and Technology, 2017), and both tests included items from the previous and next grade levels to avoid potential floor or ceiling effects. Therefore, each test consisted of 15 items for children in Grade 1 (ten characters for Grade 1 and five characters for Grade 2) and 20 items for children in Grades 2 to 6 (five characters for the previous grade level, ten characters for the corresponding grade level, and five characters for the next grade level; for Grade 6 children, ten characters each for Grades 5 and 6 were used; see Appendix H).

Both the online and paper-based tests followed the same procedure. Specifically, in both tests, children were presented with a short sentence written in Hiragana and asked to read the sentence and to write the Kanji character for the word indicated by an underline on the answer canvas on a web browser (see Fig. 1) or on a paper with numbered spaces. A child’s score in each test was the number of correct answers (max =15 for Grade 1 and 20 for Grades 2 to 6). Two trained human raters with expertise in literacy assessment independently scored all responses (*N* = 4885) as correct/incorrect based on the criteria used in the national survey (Synthetic Research Institute of Elementary Education, 2005, pp. 59–62). The initial interrater agreement was .95, and where disagreements existed, the raters discussed their scorings and came to an agreement on the score.

Importantly, in addition to the accuracy measure, OAHaS automatically recorded several response time measures (e.g., total response time, latency, duration, pause time). Here, we report the results on *writing* *latency* (defined as the time from stimulus onset to the start of writing) and *writing duration* (defined as the time from the start of the first stroke to the end of the final stroke) as indicators of children’s handwriting fluency (Asselborn et al., 2018; Rosenblum et al., 2004). Given that the number of strokes of the Kanji characters used in OAHaS varied from 1 (“一”) to 17 (“優”), both writing fluency measures were divided by the number of strokes and then used in the analysis.

Both tests were administered in the middle of the school year (November; in Japan, the school year starts in April and ends in March). All children were tested as a group by trained research assistants in their respective classrooms. The children were first tested on the online test (OAHaS) using the tablet computers installed in the school (Surface Go 2, Microsoft); they used an electronic pen attached to the computers to complete the test. The children were then tested on the traditional paper-based test on a separate day. Each test took approximately 15 min to complete.

#### Statistical analysis

To examine the psychometric property (i.e., internal consistency and validity) of OAHaS, Cronbach’s alpha reliability coefficients were calculated separately for each grade. Next, to evaluate convergent validity, the correlations between the scores assessed by OAHaS and the paper-based test were calculated for each grade. Finally, to examine the potential utility of handwriting fluency measures (writing latency and duration) assessed with OAHaS, two generalized linear models (GLMs) were fitted. In particular, we examined (1) whether the child’s grade influenced writing fluency and duration after controlling for the child’s gender and writing accuracy, and (2) whether writing accuracy influenced writing latency and duration after controlling for the child’s grade and gender. In previous studies, handwriting fluency has often been assessed using rather crude estimation measures (e.g., one-minute handwriting assessments; Kim et al., 2014; Skar et al., 2022; Wagner et al., 2011). In contrast, OAHaS allows us to simultaneously assess handwriting accuracy and fluency for each item with increased precision. By specifically investigating these two research questions, we sought to provide examples of how we can potentially use and explore these fluency measures (i.e., latency and duration) for research and practice in handwriting development. All analyses were performed using R (R Core Team, 2024). All data and analysis codes are available at https://osf.io/gver2/.

### Results

Descriptive statistics for OAHaS and the paper-based test are shown in Table 4. The skewness and kurtosis values were all in the acceptable range (Kline, 2023). Cronbach’s alpha reliability coefficients indicated that OAHaS was highly reliable in all grades (αs = .77–.90) except Grade 3 (α = .53). It should be noted, however, that the reliability coefficients for the paper-based test were very similar for all grades, including Grade 3, indicating that the relatively lower internal consistency in Grade 3 was unlikely to be due to the test format.

**Table 4:** Descriptive statistics for OAHaS and the paper-based test

Measure	*n*	M	SD	Range	Skew	Kurt	α
OAHaS							
Grade 1	35	5.71	1.99	2–13	0.99	3.38	.82
Grade 2	45	12.51	2.83	6–19	0.09	– 0.15	.77
Grade 3	36	10.83	1.81	7–14	– 0.32	– 1.02	.53
Grade 4	42	9.48	3.21	3–17	0.40	– 0.22	.79
Grade 5	48	11.40	4.65	2–20	– 0.13	– 0.80	.88
Grade 6	43	13.42	4.72	1–20	– 1.03	0.41	.90
Paper-based test							
Grade 1	35	6.31	2.32	0–14	– 0.08	3.70	.84
Grade 2	46	11.85	2.32	6–18	0.15	0.98	.78
Grade 3	37	11.68	2.30	4–16	– 0.72	1.44	.53
Grade 4	44	10.80	3.78	2–18	– 0.15	– 0.67	.81
Grade 5	50	12.14	4.22	0–20	– 0.51	0.53	.86
Grade 6	43	13.05	4.86	1–20	– 0.92	0.13	.88

*Note.* Skew = skewness; Kurt = kurtosis

**Fig. 4:**
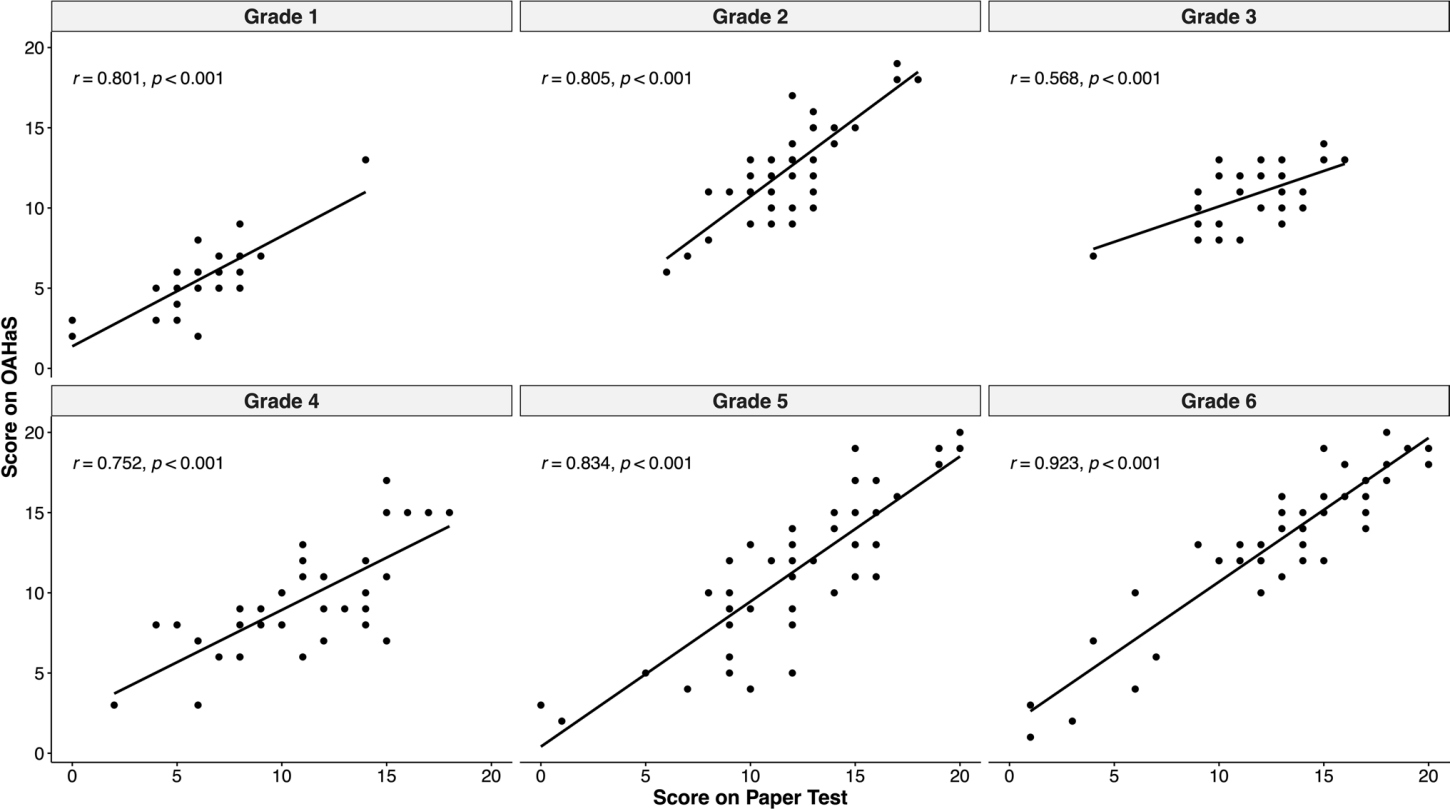
Scatter plots for the scores on OAHaS and the paper-based test using different items sets

Figure 4 shows the correlation coefficients and scatter plots for the scores on OAHaS and the paper-based test. The correlations between the two tests were high for all grades (*r*s = .75–.92, *p*s < .001) and moderate for Grade 3 (*r* = .57, *p* < .001). The highest correlation was found in Grade 6 (*r* = .92, *p* < .001). In addition, the correlation between the two tests for the entire sample was *r* = .86, *p* < .001. Taken together, these results provided behavioral evidence for the good overall reliability and convergent validity of OAHaS.

**Fig. 5:**
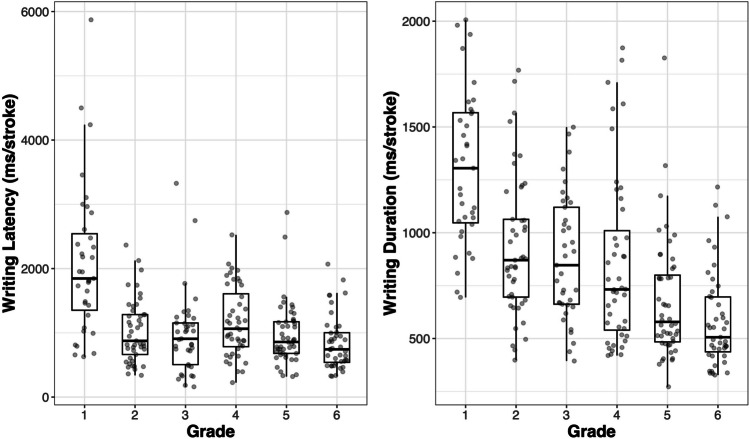
Box plots for the writing latency and duration for each grade

Finally, Fig. 5 shows the box plots for writing latency and duration for each grade, and Table 5 shows the results of the GLMs predicting writing latency and duration. As shown in Fig. 5, there was a great deal of variability in the two measures of writing fluency, both within and between grades. The results of the GLMs (Table 5) showed that children’s grade was uniquely associated with both writing latency (β = – .26, *p* < .001) and duration (β = – .57, *p* < .001), suggesting that writing fluency improves with grade. On the other hand, children’s gender was only associated with writing duration (β = – .26, *p* = . 007), with boys writing relatively faster than girls. In addition, writing accuracy was significantly associated with both writing latency (β = – .29, *p* < . 001) and duration (β = – .13, *p* = .010). These results suggest that children with higher accuracy can start writing faster and take less time to complete the writing process, reflecting the close relationship between writing accuracy and fluency.

**Table 5:** The results of the generalized linear models for writing duration and latency

Predictor	Estimate	SE	β	SE	*p*
***DV: Writing latency***					
(Intercept)	2092.20	132.85	– 0.01	0.08	< .001
Grade	– 115.09	27.19	– 0.26	0.06	< .001
Gender^a^	19.43	85.15	0.03	0.11	.820
Writing accuracy	– 50.83	10.66	– 0.29	0.06	< .001
*R*^2^	.21				
***DV: Writing duration***					
(Intercept)	1399.71	54.50	0.14	0.07	< .001
Grade	– 118.40	10.88	– 0.57	0.05	< .001
Gender^a^	– 93.06	34.44	– 0.26	0.10	.007
Writing accuracy	– 11.25	4.36	– 0.13	0.05	.010
*R*^2^	.42				

*Note.* ^a^ Coded as 0 = girls and 1 = boys

### Discussion

In the present studies, we developed a self-administered, browser-based handwriting test application (Online Assessment of Handwriting and Spelling: OAHaS) for Japanese Kanji (Study 1) and evaluated its reliability and validity through behavioral validation with data from primary school children (Study 2). We implemented an automated scoring function in OAHaS by using Convolutional Neural Network (CNN) models for image classification. The results showed first that the automated scoring of the test achieved high recall (97.98% to 98.69%) and specificity (83.03% to 84.44%), as well as high agreement with manual scoring (94.87% to 95.41%). In addition, OAHaS showed good reliability and validity for primary school children across grades (except for Grade 3), establishing a feasible, reliable, and valid platform for handwriting assessment in Japanese Kanji. In the following, we first discuss the advantages of using the CNN models for automated scoring and then discuss the psychometric properties and practical utility of the browser-based handwriting assessment.

#### Automated scoring using convolutional neural network (CNN) models

To implement an automated evaluation of handwriting responses, we applied three CNN models, namely Xception, Inception V3, and ResNet50. We trained them with different datasets of handwriting samples to examine the robustness of image classification based on extracted features. All CNN models exhibited highly accurate classification results, with over 95% accuracy across models. Compared to previous CNN studies on handwriting recognition in Japanese (Ly et al., 2017) and other languages (Chinese: Xiu et al., 2019; Bangla: Majid & Smith, 2019; English: Corbillé et al., 2020), our automated scoring function achieved a comparable or higher accuracy for the classification.

Notably, the scoring accuracy of our models showed superior performance even when compared to that of Google’s handwriting recognition service. In particular, among the key metrics (accuracy, precision, F-measure, and specificity), the difference in specificity was particularly pronounced (up to 16.97% higher compared to scoring using Google’s recognition service). Specificity reflects the model’s ability to accurately identify true negatives (i.e., characters that are truly incorrect or truly non-Kanji characters). This indicates that our CNN models were better at filtering out incorrectly written or non-Kanji characters than Google’s handwriting recognition service. This is particularly important for scoring purposes because high specificity ensures that the model not only recognizes correct characters but also effectively rejects incorrect ones, which is essential for scoring accuracy in handwriting assessments.

It should be noted that although Google’s recognition service had higher recall scores (98.50% to 99.66%), it likely ran the risk of over-identifying characters (i.e., incorrectly identifying non-Kanji characters as correct), which could have led to a higher rate of false positives. This may explain the poorer specificity scores of Google’s recognition service; in other words, although it was good at identifying true positives, this appears to have come at the expense of lower specificity (Saito & Rehmsmeier, 2015). In contrast, our CNN models showed a more balanced performance with high accuracy, precision, specificity, and F-measure, suggesting a more reliable overall classification. Overall, compared to Google’s handwriting recognition service, our models offer more balanced performance across all the metrics, especially in distinguishing between correct and incorrect handwriting, making them more suitable for handwriting classification for scoring purposes. This is a noteworthy achievement, particularly given the relatively small number of handwriting samples used for model development (the total number of images in the three datasets was 10,578).

A key factor contributing to the high accuracy of our CNN models may be the pre-extraction of features, which has been done automatically by the models with the advanced algorithms. CNN models have been shown to outperform conventional feature extraction approaches in discerning handwriting representations, as they can learn the informative features directly from the data rather than manual feature extraction (Jasira et al., 2023; Morera et al., 2018). This is exemplified by the high classification performance of our models for Japanese Kanji characters (Table 3). Interestingly, our CNN models showed higher performance when they were trained not only on correctly written characters (Dataset 1) but also on characters that contained common errors (Datasets 2 and 3). This indicates that at least in the context of handwriting assessment, including common errors in model training datasets is a crucial step in improving scoring accuracy. Our results add to the fast-growing literature using an advanced CNN architecture in handwriting classification (Isa et al., 2021; Kartika et al., 2023; Morera et al., 2018; Rahmanian & Shayegan, 2021; Rosli et al., 2021) by extending it to Japanese Kanji characters, which contain intricate spatial constructions (Chang et al., 2018) that would be difficult to encode using traditional manual feature extraction approaches.

#### Psychometric properties and practical utility

In Study 2, we examined the psychometric properties (i.e., reliability and validity) of OAHaS in behavioral validation with data from Japanese primary school children. Our results demonstrated that the online test exhibited good reliability and validity across grades. Specifically, OAHaS showed high internal consistency (α = .77–.90) in all grades, except Grade 3 (α = .53). In addition, children’s performance on the online test was strongly correlated with that on a paper-based test using a different set of items (*r*s = .86 for the entire sample and .57–.92 for each grade).

To date, only a handful of online assessments of language and literacy have established reliability and validity (e.g., Hulme et al., 2024; Sobers et al., 2023; Yeatman et al., 2021). For example, Yeatman et al. (2021) developed a browser-based word reading assessment with a strong correlation (*r* = .86) with a standardized paper-based reading test (Letter-Word Identification from Woodcock-Johnson; Woodcock et al., 2001). Similarly, Sobers et al. (2023) reported that their cell phone-based remote assessments evaluating several aspects of language and literacy showed moderate to strong correlations (*r*s = .35–.80) with traditional paper-based assessments. While our studies examined handwriting skills, the correlations between OAHaS and the paper-based writing test were comparable to those reported for these online language and reading assessments. Importantly, OAHaS contributes to this line of research by demonstrating the sound validity of the automated, browser-based assessment of handwriting, opening a new avenue for research and practice on handwriting and spelling.

Our results further demonstrated the practical utility of writing fluency measures assessed automatically by OAHaS. For example, we found that writing latency and duration decreased significantly with grade, and boys wrote relatively faster than girls. Additionally, children with higher accuracy started writing answers more quickly and took less time to complete writing (see Table 5). To our knowledge, this is the first online assessment that measures handwriting accuracy and fluency automatically and simultaneously. The browser-based handwriting assessment developed here not only provides a rapid, reliable, and valid assessment of handwriting skills but also provides researchers and practitioners with valuable insights into the complex processes of handwriting. OAHaS enables us to capture subtle writing patterns that cannot be captured by the human eye, including latency, duration, and other temporal metrics. This, in turn, can offer insights into handwriting processes and a deeper understanding of children’s writing development that traditional paper-based assessment cannot reveal (e.g., Ho et al., 2007; Wilkinson & Robertson, 2006).

#### Limitations and future research

Some limitations of our studies are worth mentioning. First, our findings can only be generalized to the language under study (Japanese Kanji) and to the ages of the participants we had in our sample (Grades 1 to 6). We can note, however, that our CNN models employed a language-independent technique that is applicable to any language and script (see e.g., Corbillé et al., 2020; Majid & Smith, 2019). Future studies should examine the applicability of our test development framework across different writing systems, including both morphographic (e.g., Chinese) and alphabetic (e.g., English) scripts.

Second, we only assessed children on handwriting skills (accuracy and fluency). Future studies should include additional measures of other cognitive skills (e.g., visual memory, speed of processing), graphomotor skills, and higher-level writing skills (e.g., transcription) to further examine the role of handwriting from a broader perspective. Finally, the number of handwriting samples in our CNN model development was relatively small. Future studies should consider using a more extensive set of diverse handwriting samples to develop CNN models, which may further increase their sensitivity to detect wider variations in the features of correct/incorrect responses.

## Conclusion

To conclude, in the present studies, we developed a self-administered, browser-based handwriting test application (Online Assessment of Handwriting and Spelling: OAHaS) for Japanese Kanji and evaluated its psychometric properties. We implemented an automated scoring function in OAHaS using Convolutional Neural Network (CNN) models for image classification. The automated scoring function achieved high classification accuracy and agreement with manual scoring. Additionally, behavioral validation showed that children’s scores on the online test were strongly correlated with their scores on a paper-based test (*r* = .86). Furthermore, our analysis suggested the practical utility of writing fluency measures (latency and duration) that are automatically captured by OAHaS. Overall, these findings add to the growing body of research developing online assessments of language and literacy by providing evidence for the reliability and validity of OAHaS as a measure of handwriting. The test enables researchers and practitioners to efficiently assess handwriting accuracy and fluency, thereby providing a rapid and informative assessment tool for research and practice on handwriting development and difficulties.

## Supplementary Information

Below is the link to the electronic supplementary material.

Supplementary file1 (DOCX 628 KB)

## Data Availability

All data and materials, including handwriting samples for model training, behavioral validation data, and testing materials, are available at https://osf.io/gver2/.

## Footnotes

1 Because of its simplicity, most Japanese children quickly master reading Hiragana within the first few months in Grade 1 (Inoue et al., 2017; Shimamura & Mikami, 1994).

2 In OAHaS, this step was replaced with our CNN models following the model development phase.

## Code availability

All source code for the application development and data analysis code for the behavioral validation are available at https://osf.io/gver2/.

The authors do not have any conflict to disclose.
