# Supplementary material for: Assessing handwriting skills in a web browser: Development and validation of an automated online test in Japanese Kanji
Source: Behav Res Methods. 2024 Dec 30;57(1):32. doi: 10.3758/s13428-024-02562-6 (PMC11685258; doi:10.3758/s13428-024-02562-6)
Supplement: Supplementary file 1 — Supplementary file1 (DOCX 628 KB) [file 13428_2024_2562_MOESM1_ESM.docx]

Appendix A

The Items Used in the Online and Paper-and-Pencil Tests

| ID | Grade^a^ | Kanji | Samples^b^ |  | ID | Grade^a^ | Kanji | Samples^b^ |  | ID | Grade^a^ | Kanji | Samples^b^ |
| --- | --- | --- | --- | --- | --- | --- | --- | --- | --- | --- | --- | --- | --- |
| 001 | 1 | 一 | 139 |  | 041 | 3 | 面 | 62 |  | 081 | 5 | 支 | 62 |
| 002 | 1 | 川 | 131 |  | 042 | 3 | 動 | 62 |  | 082 | 5 | 賀 | 58 |
| 003 | 1 | 口 | 123 |  | 043 | 3 | 丁 | 65 |  | 083 | 5 | 罪 | 58 |
| 004 | 1 | 山 | 115 |  | 044 | 3 | 係 | 61 |  | 084 | 5 | 豊 | 55 |
| 005 | 1 | 女 | 112 |  | 045 | 3 | 使 | 59 |  | 085 | 5 | 述 | 60 |
| 006 | 1 | 水 | 108 |  | 046 | 3 | 表 | 58 |  | 086 | 5 | 能 | 59 |
| 007 | 1 | 花 | 99 |  | 047 | 3 | 集 | 53 |  | 087 | 5 | 格 | 60 |
| 008 | 1 | 子 | 97 |  | 048 | 3 | 秒 | 56 |  | 088 | 5 | 雑 | 58 |
| 009 | 1 | 大 | 94 |  | 049 | 3 | 定 | 62 |  | 089 | 5 | 移 | 61 |
| 010 | 1 | 月 | 88 |  | 050 | 3 | 取 | 49 |  | 090 | 5 | 責 | 60 |
| 011 | 1 | 赤 | 85 |  | 051 | 3 | 暗 | 60 |  | 091 | 5 | 演 | 73 |
| 012 | 1 | 草 | 78 |  | 052 | 3 | 暑 | 60 |  | 092 | 5 | 刊 | 76 |
| 013 | 1 | 正 | 77 |  | 053 | 3 | 筆 | 48 |  | 093 | 5 | 示 | 74 |
| 014 | 1 | 校 | 72 |  | 054 | 3 | 飲 | 64 |  | 094 | 5 | 久 | 72 |
| 015 | 1 | 足 | 69 |  | 055 | 3 | 代 | 62 |  | 095 | 5 | 解 | 60 |
| 016 | 1 | 村 | 64 |  | 056 | 3 | 打 | 63 |  | 096 | 5 | 確 | 63 |
| 017 | 1 | 百 | 69 |  | 057 | 3 | 緑 | 58 |  | 097 | 5 | 構 | 64 |
| 018 | 1 | 先 | 67 |  | 058 | 3 | 根 | 60 |  | 098 | 5 | 寄 | 65 |
| 019 | 1 | 虫 | 64 |  | 059 | 3 | 等 | 64 |  | 099 | 5 | 衛 | 63 |
| 020 | 1 | 入 | 67 |  | 060 | 3 | 港 | 63 |  | 100 | 5 | 制 | 64 |
| 021 | 2 | 広 | 65 |  | 061 | 4 | 争 | 27 |  | 101 | 6 | 宝 | 36 |
| 022 | 2 | 書 | 57 |  | 062 | 4 | 夫 | 32 |  | 102 | 6 | 呼 | 37 |
| 023 | 2 | 外 | 59 |  | 063 | 4 | 不 | 29 |  | 103 | 6 | 骨 | 33 |
| 024 | 2 | 答 | 61 |  | 064 | 4 | 冷 | 28 |  | 104 | 6 | 難 | 31 |
| 025 | 2 | 少 | 60 |  | 065 | 4 | 変 | 28 |  | 105 | 6 | 優 | 28 |
| 026 | 2 | 行 | 143 |  | 066 | 4 | 億 | 24 |  | 106 | 6 | 聖 | 26 |
| 027 | 2 | 家 | 54 |  | 067 | 4 | 束 | 28 |  | 107 | 6 | 乱 | 35 |
| 028 | 2 | 台 | 59 |  | 068 | 4 | 機 | 27 |  | 108 | 6 | 痛 | 32 |
| 029 | 2 | 作 | 58 |  | 069 | 4 | 梅 | 27 |  | 109 | 6 | 蚕 | 38 |
| 030 | 2 | 雲 | 50 |  | 070 | 4 | 健 | 26 |  | 110 | 6 | 郷 | 35 |
| 031 | 2 | 紙 | 79 |  | 071 | 4 | 隊 | 55 |  | 111 | 6 | 宣 | 36 |
| 032 | 2 | 晴 | 74 |  | 072 | 4 | 良 | 57 |  | 112 | 6 | 傷 | 35 |
| 033 | 2 | 東 | 85 |  | 073 | 4 | 候 | 57 |  | 113 | 6 | 臨 | 33 |
| 034 | 2 | 元 | 61 |  | 074 | 4 | 昨 | 60 |  | 114 | 6 | 視 | 38 |
| 035 | 2 | 行 | 143 |  | 075 | 4 | 借 | 57 |  | 115 | 6 | 律 | 35 |
| 036 | 2 | 通 | 61 |  | 076 | 4 | 望 | 60 |  | 116 | 6 | 看 | 30 |
| 037 | 2 | 黄 | 70 |  | 077 | 4 | 挙 | 61 |  | 117 | 6 | 頂 | 34 |
| 038 | 2 | 矢 | 83 |  | 078 | 4 | 票 | 59 |  | 118 | 6 | 厳 | 27 |
| 039 | 2 | 頭 | 76 |  | 079 | 4 | 腸 | 53 |  | 119 | 6 | 陛 | 36 |
| 040 | 2 | 合 | 85 |  | 080 | 4 | 刷 | 56 |  | 120 | 6 | 供 | 35 |

*Note.* ^a^ “Grade” indicates the grade in which children learn the character according to the national curriculum (Ministry of Education, Culture, Sports, Science and Technology, 2017). ^b^ “Samples” indicate the number of handwriting samples collected using OAHaS.

Appendix B

Example Images of Characters with Orthographic Errors (Dataset 2)

| ID | Kanji | Grade | *N* | Example Image |
| --- | --- | --- | --- | --- |
| 017-NG1 | 百 | 1 | 69 | 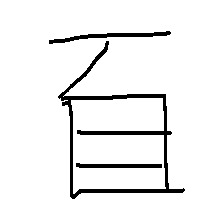 |
| 025-NG1 | 少 | 2 | 60 | 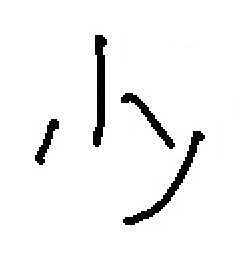 |
| 037-NG1 | 黄 | 2 | 70 | 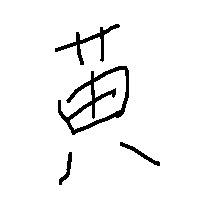 |
| 037-NG2 | 黄 | 2 | 70 | 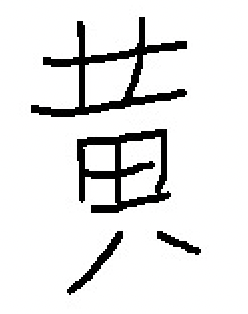 |
| 045-NG1 | 使 | 3 | 59 | 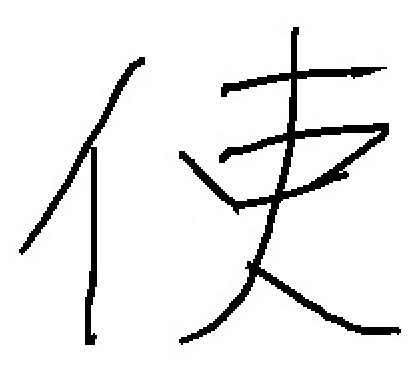 |
| 051-NG1 | 暗 | 3 | 60 | 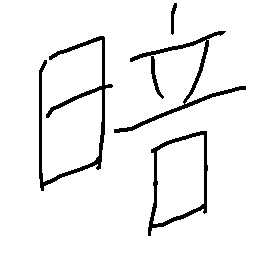 |
| 057-NG1 | 緑 | 3 | 58 | 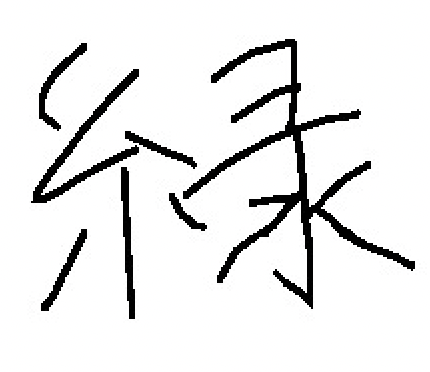 |
| 077-NG1 | 挙 | 4 | 61 | 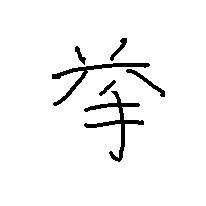 |
| 091-NG1 | 演 | 5 | 73 | 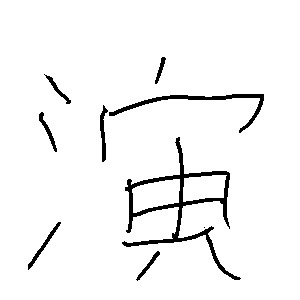 |
| 095-NG1 | 解 | 5 | 60 | 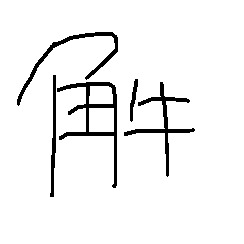 |
| 095-NG2 | 解 | 5 | 60 | 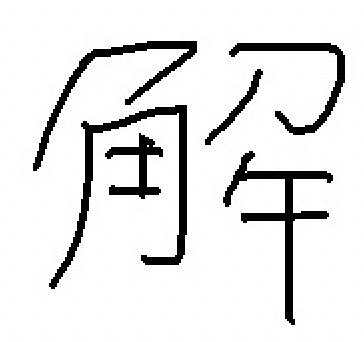 |
| 097-NG1 | 構 | 5 | 64 | 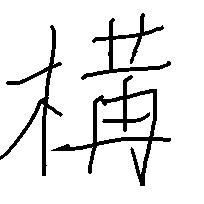 |
| 099-NG1 | 衛 | 5 | 63 | 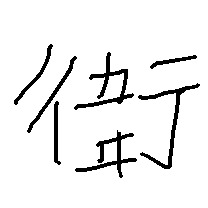 |
| 101-NG1 | 宝 | 6 | 36 | 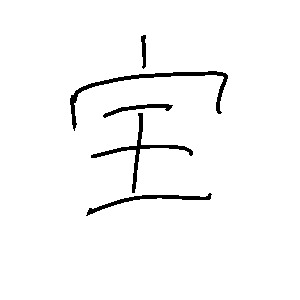 |
| 103-NG1 | 骨 | 6 | 33 | 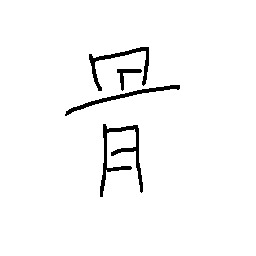 |
| 105-NG1 | 優 | 6 | 28 | 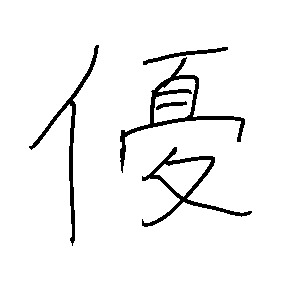 |
| 105-NG2 | 優 | 6 | 28 | 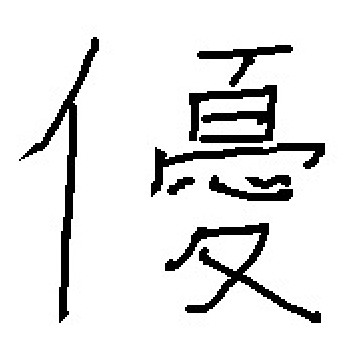 |
| 111-NG1 | 宣 | 6 | 36 | 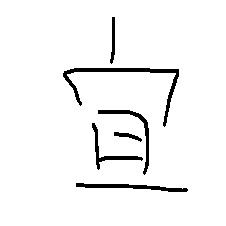 |
| 115-NG1 | 律 | 6 | 35 | 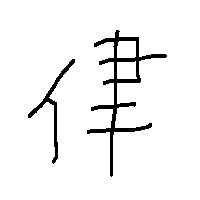 |
| Total |  |  | 1023 |  |

*Note.* All example images are incorrect Kanji characters that contain orthographic errors (e.g., extra/missing strokes or stroke intersections). The full dataset is available at <https://osf.io/gver2/>.

Appendix C

Example Images of Characters with Semantic/Homophone Errors (Dataset 3)

| Unicode | Kanji | Grade | *N* |
| --- | --- | --- | --- |
| 0x4e09 | 三 | 1 | 140 |
| 0x5c0f | 小 | 1 | 155 |
| 0x65e5 | 日 | 1 | 153 |
| 0x4ea4 | 交 | 2 | 138 |
| 0x5200 | 刀 | 2 | 152 |
| 0x5207 | 切 | 2 | 129 |
| 0x6b62 | 止 | 2 | 150 |
| 0x7dda | 線 | 2 | 108 |
| 0x9854 | 顔 | 2 | 75 |
| 0x5316 | 化 | 3 | 191 |
| 0x554f | 問 | 3 | 181 |
| 0x967d | 陽 | 3 | 54 |
| 0x968e | 階 | 3 | 69 |
| 0x5404 | 各 | 4 | 147 |
| 0x6c42 | 求 | 4 | 117 |
| 0x9678 | 陸 | 4 | 114 |
| 0x8ff7 | 迷 | 5 | 111 |
| 0x9632 | 防 | 5 | 119 |
| 0x6d25 | 津 | - | 73 |
| 0x6ed1 | 滑 | - | 46 |
| Total |  |  | 2422 |

*Note.* All Kanji characters are existent characters that do not match the word context given in the test items. The full dataset is available at <https://osf.io/gver2/>.

Appendix D

Scanned Handwriting Samples for the Model Validation

| Item | Grade | Kanji |  | g1f | |  | g2s | |
| --- | --- | --- | --- | --- | --- | --- | --- | --- |
|  |  |  |  | Correct | Incorrect |  | Correct | Incorrect |
| 001 | 1 | 一 |  | 153 |  |  |  |  |
| 002 | 1 | 川 |  | 147 |  |  | 135 |  |
| 003 | 1 | 口 |  | 148 | 1 |  |  |  |
| 004 | 1 | 山 |  | 139 | 1 |  |  |  |
| 005 | 1 | 女 |  | 95 | 3 |  | 133 | 1 |
| 006 | 1 | 水 |  | 140 | 1 |  |  |  |
| 007 | 1 | 花 |  | 77 | 5 |  | 134 | 1 |
| 008 | 1 | 子 |  | 106 | 2 |  | 132 | 1 |
| 009 | 1 | 大 |  | 134 | 2 |  |  |  |
| 010 | 1 | 月 |  | 120 | 2 |  | 143 | 1 |
| 011 | 1 | 赤 |  | 46 | 8 |  | 119 | 12 |
| 012 | 1 | 草 |  | 22 | 7 |  | 108 | 15 |
| 013 | 1 | 正 |  | 92 | 10 |  | 115 | 9 |
| 014 | 1 | 校 |  | 68 | 5 |  | 122 | 4 |
| 015 | 1 | 足 |  | 59 | 11 |  | 44 | 10 |
| 016 | 1 | 村 |  | 16 | 9 |  | 90 | 14 |
| 017 | 1 | 百 |  | 65 | 22 |  | 110 | 12 |
| 018 | 1 | 先 |  | 29 | 24 |  | 102 | 12 |
| 019 | 1 | 虫 |  | 63 | 11 |  | 103 | 11 |
| 020 | 1 | 入 |  | 55 | 15 |  | 42 | 14 |
| 021 | 2 | 広 |  | 4 | 2 |  | 38 | 3 |
| 022 | 2 | 書 |  | 3 | 11 |  | 102 | 9 |
| 023 | 2 | 外 |  | 8 | 2 |  | 37 | 13 |
| 024 | 2 | 答 |  | 3 | 2 |  | 8 | 6 |
| 025 | 2 | 少 |  | 3 | 2 |  | 18 | 12 |
| 026 | 2 | 行 |  | 4 | 7 |  | 71 | 20 |
| 027 | 2 | 家 |  | 2 | 4 |  | 12 | 10 |
| 028 | 2 | 台 |  | 2 | 21 |  | 9 | 51 |
| 029 | 2 | 作 |  | 2 | 9 |  | 15 | 10 |
| 030 | 2 | 雲 |  | 2 | 5 |  | 5 | 2 |
| 031 | 2 | 紙 |  | 2 | 1 |  | 67 | 11 |
| 032 | 2 | 晴 |  | 1 | 1 |  | 74 | 9 |
| 033 | 2 | 東 |  | 1 | 2 |  | 8 | 3 |
| 034 | 2 | 元 |  |  |  |  | 5 | 3 |
| 035 | 2 | 行 |  | 1 |  |  | 3 | 1 |
| 036 | 2 | 通 |  | 1 | 1 |  | 2 | 2 |
| 037 | 2 | 黄 |  | 1 | 1 |  | 48 | 15 |
| 038 | 2 | 矢 |  | 3 | 1 |  | 8 | 10 |
| 039 | 2 | 頭 |  | 2 | 1 |  | 6 | 6 |
| 040 | 2 | 合 |  |  |  |  | 2 |  |
| 041 | 3 | 面 |  |  | 1 |  | 5 | 6 |
| 042 | 3 | 動 |  |  |  |  | 1 |  |
| 043 | 3 | 丁 |  |  | 1 |  | 4 | 3 |
| 044 | 3 | 係 |  |  |  |  | 1 | 1 |
| 045 | 3 | 使 |  | 1 |  |  | 1 |  |
| 046 | 3 | 表 |  |  |  |  |  | 1 |
| 047 | 3 | 集 |  |  |  |  | 3 | 1 |
| 048 | 3 | 秒 |  |  | 1 |  |  | 1 |
| 049 | 3 | 定 |  |  |  |  | 1 | 1 |
| 050 | 3 | 取 |  |  |  |  |  |  |
| 051 | 3 | 暗 |  |  |  |  | 1 |  |
| 052 | 3 | 暑 |  |  |  |  | 1 | 1 |
| 053 | 3 | 筆 |  |  |  |  | 2 |  |
| 054 | 3 | 飲 |  |  |  |  |  |  |
| 055 | 3 | 代 |  |  |  |  |  |  |
| 056 | 3 | 打 |  |  |  |  | 1 |  |
| 057 | 3 | 緑 |  |  |  |  |  |  |
| 058 | 3 | 根 |  |  |  |  |  | 2 |
| 059 | 3 | 等 |  |  |  |  |  |  |
| 060 | 3 | 港 |  |  |  |  |  |  |

*Note.* The total number of samples was 7,128. The full dataset is available at <https://osf.io/gver2/>.

Appendix E

Discriminant Accuracy, Precision, Recall, F-measure, and Specificity Values for Google’s Handwriting Recognition Service

| Criterion Rank |  | Match | | |  | Mismatch | | |  |  |  |  |  |  |
| --- | --- | --- | --- | --- | --- | --- | --- | --- | --- | --- | --- | --- | --- | --- |
|  |  | TP | TN | Total |  | FN | FP | Total |  | Accuracy | Precision | Recall | F-measure | Specificity |
| 1 |  | 2628 | 544 | 3172 |  | 40 | 163 | 203 |  | 93.99 | 94.16 | 98.50 | 96.28 | 76.94 |
| 2 |  | 2649 | 509 | 3158 |  | 19 | 198 | 217 |  | 93.57 | 93.05 | 99.29 | 96.07 | 71.99 |
| 3 |  | 2652 | 491 | 3143 |  | 16 | 216 | 232 |  | 93.13 | 92.47 | 99.40 | 95.81 | 69.45 |
| 4 |  | 2654 | 484 | 3138 |  | 14 | 223 | 237 |  | 92.98 | 92.25 | 99.48 | 95.73 | 68.46 |
| 5 |  | 2659 | 477 | 3136 |  | 9 | 221 | 230 |  | 92.92 | 92.04 | 99.66 | 95.70 | 67.47 |

*Note.* Criterion rank represents the cutoff used to determine correctness among the five candidates returned by the Google service. For example, if the criterion rank is 3, the answer will be considered correct if the first three candidates contain the target kanji character. TP = true positive; TN = true negative; FN = false negative; FP = false positive.

Appendix F

The Models’ Performance for an Independent Handwriting Sample from Grade 1 Children (g1f)

|  |  | Match | | |  | Mismatch | | |  |  |  |  |  |  |
| --- | --- | --- | --- | --- | --- | --- | --- | --- | --- | --- | --- | --- | --- | --- |
| Model and Dataset |  | TP | TN | Total |  | FN | FP | Total |  | Accuracy | Precision | Recall | F-measure | Specificity |
| Xception |  |  |  |  |  |  |  |  |  |  |  |  |  |  |
| Dataset 1 |  | 1790 | 145 | 1935 |  | 30 | 70 | 100 |  | 95.09 | 96.24 | 98.35 | 97.28 | 67.44 |
| Dataset 1+2 |  | 1794 | 156 | 1950 |  | 26 | 59 | 85 |  | 95.82 | 96.82 | 98.57 | 97.69 | 72.56 |
| Dataset 1+3 |  | 1799 | 137 | 1936 |  | 21 | 78 | 99 |  | 95.14 | 95.84 | 98.85 | 97.32 | 63.72 |
| Dataset 1+2+3 |  | 1798 | 165 | 1963 |  | 22 | 50 | 72 |  | 96.46 | 97.29 | 98.79 | 98.04 | 76.74 |
| Inception V3 |  |  |  |  |  |  |  |  |  |  |  |  |  |  |
| Dataset 1 |  | 1674 | 147 | 1821 |  | 146 | 68 | 214 |  | 89.48 | 96.10 | 91.98 | 93.99 | 68.37 |
| Dataset 1+2 |  | 1731 | 169 | 1900 |  | 89 | 46 | 135 |  | 93.37 | 97.41 | 95.11 | 96.25 | 78.60 |
| Dataset 1+3 |  | 1788 | 137 | 1925 |  | 32 | 78 | 110 |  | 94.59 | 95.82 | 98.24 | 97.02 | 63.72 |
| Dataset 1+2+3 |  | 1778 | 174 | 1952 |  | 42 | 41 | 83 |  | 95.92 | 97.75 | 97.69 | 97.72 | 80.93 |
| ResNet50 |  |  |  |  |  |  |  |  |  |  |  |  |  |  |
| Dataset 1 |  | 1780 | 135 | 1915 |  | 40 | 80 | 120 |  | 94.10 | 95.70 | 97.80 | 96.74 | 62.79 |
| Dataset 1+2 |  | 1784 | 157 | 1941 |  | 36 | 58 | 94 |  | 95.38 | 96.85 | 98.02 | 97.43 | 73.02 |
| Dataset 1+3 |  | 1784 | 136 | 1920 |  | 36 | 79 | 115 |  | 94.35 | 95.76 | 98.02 | 96.88 | 63.26 |
| Dataset 1+2+3 |  | 1794 | 157 | 1951 |  | 26 | 58 | 84 |  | 95.87 | 96.87 | 98.57 | 97.71 | 73.02 |

*Note.* The first column indicates the base model and the datasets used to develop the model. TP = true positive; TN = true negative; FN = false negative; FP = false positive.

Appendix G

The Models’ Performance for an Independent Handwriting Sample from Grade 2 Children (g2s)

|  |  | Match | | |  | Mismatch | | |  |  |  |  |  |  |
| --- | --- | --- | --- | --- | --- | --- | --- | --- | --- | --- | --- | --- | --- | --- |
| Model and Dataset |  | TP | TN | Total |  | FN | FP | Total |  | Accuracy | Precision | Recall | F-measure | Specificity |
| Xception |  |  |  |  |  |  |  |  |  |  |  |  |  |  |
| Dataset 1 |  | 2150 | 207 | 2357 |  | 41 | 123 | 164 |  | 93.49 | 94.59 | 98.13 | 96.33 | 62.73 |
| Dataset 1+2 |  | 2172 | 227 | 2399 |  | 19 | 103 | 122 |  | 95.16 | 95.47 | 99.13 | 97.27 | 68.79 |
| Dataset 1+3 |  | 2178 | 196 | 2374 |  | 13 | 134 | 147 |  | 94.17 | 94.20 | 99.41 | 96.74 | 59.39 |
| Dataset 1+2+3 |  | 2163 | 221 | 2384 |  | 28 | 109 | 137 |  | 94.57 | 95.20 | 98.72 | 96.93 | 66.97 |
| Inception V3 |  |  |  |  |  |  |  |  |  |  |  |  |  |  |
| Dataset 1 |  | 2031 | 224 | 2255 |  | 160 | 106 | 266 |  | 89.45 | 95.04 | 92.70 | 93.85 | 67.88 |
| Dataset 1+2 |  | 2054 | 228 | 2282 |  | 137 | 102 | 239 |  | 90.52 | 95.27 | 93.75 | 94.50 | 69.09 |
| Dataset 1+3 |  | 2141 | 198 | 2339 |  | 50 | 132 | 182 |  | 92.78 | 94.19 | 97.72 | 95.92 | 60.00 |
| Dataset 1+2+3 |  | 2132 | 222 | 2354 |  | 59 | 108 | 167 |  | 93.38 | 95.18 | 97.31 | 96.23 | 67.27 |
| ResNet50 |  |  |  |  |  |  |  |  |  |  |  |  |  |  |
| Dataset 1 |  | 2148 | 197 | 2345 |  | 43 | 133 | 176 |  | 93.02 | 94.17 | 98.04 | 96.06 | 59.70 |
| Dataset 1+2 |  | 2145 | 215 | 2360 |  | 46 | 115 | 161 |  | 93.61 | 94.91 | 97.90 | 96.38 | 65.15 |
| Dataset 1+3 |  | 2143 | 195 | 2338 |  | 48 | 135 | 183 |  | 92.74 | 94.07 | 97.81 | 95.91 | 59.09 |
| Dataset 1+2+3 |  | 2130 | 209 | 2339 |  | 61 | 121 | 182 |  | 92.78 | 94.62 | 97.22 | 95.90 | 63.33 |

*Note.* The first column indicates the base model and the datasets used to develop the model. TP = true positive; TN = true negative; FN = false negative; FP = false positive.

Appendix H

The Items Used in the Online Handwriting Test (OAHaS)

| Items | Kanji | Grade^a^ | Items used for each grade | | | | | |  | Answers | | |
| --- | --- | --- | --- | --- | --- | --- | --- | --- | --- | --- | --- | --- |
|  |  |  | 1 | 2 | 3 | 4 | 5 | 6 |  | Correct | Incorrect | No response |
| 001 | 一 | 1 | ✓ |  |  |  |  |  |  | 34 | 1 | 0 |
| 003 | 口 | 1 | ✓ |  |  |  |  |  |  | 26 | 1 | 8 |
| 005 | 女 | 1 | ✓ |  |  |  |  |  |  | 29 | 2 | 4 |
| 007 | 花 | 1 | ✓ |  |  |  |  |  |  | 4 | 1 | 30 |
| 009 | 大 | 1 | ✓ |  |  |  |  |  |  | 35 | 0 | 0 |
| 011 | 赤 | 1 | ✓ | ✓ |  |  |  |  |  | 44 | 4 | 32 |
| 013 | 正 | 1 | ✓ | ✓ |  |  |  |  |  | 70 | 5 | 5 |
| 015 | 足 | 1 | ✓ | ✓ |  |  |  |  |  | 47 | 0 | 33 |
| 017 | 百 | 1 | ✓ | ✓ |  |  |  |  |  | 38 | 15 | 27 |
| 019 | 虫 | 1 | ✓ | ✓ |  |  |  |  |  | 72 | 5 | 3 |
| 021 | 広 | 2 | ✓ | ✓ |  |  |  |  |  | 42 | 2 | 36 |
| 023 | 外 | 2 | ✓ | ✓ |  |  |  |  |  | 41 | 3 | 36 |
| 025 | 少 | 2 | ✓ | ✓ |  |  |  |  |  | 36 | 5 | 39 |
| 027 | 家 | 2 | ✓ | ✓ |  |  |  |  |  | 44 | 2 | 34 |
| 029 | 作 | 2 | ✓ | ✓ |  |  |  |  |  | 33 | 5 | 42 |
| 031 | 紙 | 2 |  | ✓ | ✓ |  |  |  |  | 79 | 1 | 2 |
| 033 | 東 | 2 |  | ✓ | ✓ |  |  |  |  | 65 | 10 | 7 |
| 035 | 行 | 2 |  | ✓ | ✓ |  |  |  |  | 47 | 3 | 32 |
| 037 | 黄 | 2 |  | ✓ | ✓ |  |  |  |  | 57 | 18 | 7 |
| 039 | 頭 | 2 |  | ✓ | ✓ |  |  |  |  | 62 | 12 | 8 |
| 041 | 面 | 3 |  | ✓ | ✓ |  |  |  |  | 39 | 6 | 37 |
| 043 | 丁 | 3 |  | ✓ | ✓ |  |  |  |  | 28 | 16 | 38 |
| 045 | 使 | 3 |  | ✓ | ✓ |  |  |  |  | 35 | 10 | 37 |
| 047 | 集 | 3 |  | ✓ | ✓ |  |  |  |  | 29 | 8 | 45 |
| 049 | 定 | 3 |  | ✓ | ✓ |  |  |  |  | 25 | 7 | 50 |
| 051 | 暗 | 3 |  |  | ✓ | ✓ |  |  |  | 69 | 11 | 1 |
| 053 | 筆 | 3 |  |  | ✓ | ✓ |  |  |  | 63 | 13 | 5 |
| 055 | 代 | 3 |  |  | ✓ | ✓ |  |  |  | 10 | 39 | 32 |
| 057 | 緑 | 3 |  |  | ✓ | ✓ |  |  |  | 54 | 22 | 5 |
| 059 | 等 | 3 |  |  | ✓ | ✓ |  |  |  | 25 | 31 | 25 |
| 061 | 争 | 4 |  |  | ✓ | ✓ |  |  |  | 34 | 6 | 41 |
| 063 | 不 | 4 |  |  | ✓ | ✓ |  |  |  | 44 | 3 | 34 |
| 065 | 変 | 4 |  |  | ✓ | ✓ |  |  |  | 39 | 12 | 30 |
| 067 | 束 | 4 |  |  | ✓ | ✓ |  |  |  | 20 | 12 | 49 |
| 069 | 梅 | 4 |  |  | ✓ | ✓ |  |  |  | 44 | 6 | 31 |
| 071 | 隊 | 4 |  |  |  | ✓ | ✓ |  |  | 51 | 24 | 17 |
| 073 | 候 | 4 |  |  |  | ✓ | ✓ |  |  | 32 | 20 | 40 |
| 075 | 借 | 4 |  |  |  | ✓ | ✓ |  |  | 58 | 11 | 23 |
| 077 | 挙 | 4 |  |  |  | ✓ | ✓ |  |  | 42 | 20 | 30 |
| 079 | 腸 | 4 |  |  |  | ✓ | ✓ |  |  | 16 | 23 | 53 |
| 081 | 支 | 5 |  |  |  | ✓ | ✓ | ✓ |  | 90 | 14 | 32 |
| 083 | 罪 | 5 |  |  |  | ✓ | ✓ | ✓ |  | 86 | 10 | 40 |
| 085 | 述 | 5 |  |  |  | ✓ | ✓ | ✓ |  | 72 | 16 | 48 |
| 087 | 格 | 5 |  |  |  | ✓ | ✓ | ✓ |  | 65 | 28 | 43 |
| 089 | 移 | 5 |  |  |  | ✓ | ✓ | ✓ |  | 73 | 24 | 39 |
| 091 | 演 | 5 |  |  |  |  | ✓ | ✓ |  | 48 | 20 | 24 |
| 093 | 示 | 5 |  |  |  |  | ✓ | ✓ |  | 56 | 16 | 20 |
| 095 | 解 | 5 |  |  |  |  | ✓ | ✓ |  | 63 | 22 | 7 |
| 097 | 構 | 5 |  |  |  |  | ✓ | ✓ |  | 51 | 23 | 18 |
| 099 | 衛 | 5 |  |  |  |  | ✓ | ✓ |  | 69 | 12 | 11 |
| 101 | 宝 | 6 |  |  |  |  | ✓ | ✓ |  | 77 | 10 | 5 |
| 103 | 骨 | 6 |  |  |  |  | ✓ | ✓ |  | 47 | 18 | 27 |
| 105 | 優 | 6 |  |  |  |  | ✓ | ✓ |  | 33 | 35 | 24 |
| 107 | 乱 | 6 |  |  |  |  | ✓ | ✓ |  | 44 | 10 | 38 |
| 109 | 蚕 | 6 |  |  |  |  | ✓ | ✓ |  | 41 | 8 | 43 |
| 111 | 宣 | 6 |  |  |  |  |  | ✓ |  | 6 | 14 | 24 |
| 113 | 臨 | 6 |  |  |  |  |  | ✓ |  | 21 | 5 | 18 |
| 115 | 律 | 6 |  |  |  |  |  | ✓ |  | 31 | 9 | 4 |
| 117 | 頂 | 6 |  |  |  |  |  | ✓ |  | 25 | 2 | 17 |
| 119 | 陛 | 6 |  |  |  |  |  | ✓ |  | 8 | 16 | 20 |

*Note.* The test consisted of 15 items for Grade 1 and 20 items for Grade 2 to Grade 6 (see the Materials and Procedure section in Study 2).

^a^ “Grade” indicates the grade level at which children learn the character according to the national curriculum (Ministry of Education, Culture, Sports, Science and Technology, 2017).
